# Systolic Ejection Murmur in a Case of Patent Ductus Arteriosus and Pulmonary Hypertension With Liver Cirrhosis

**DOI:** 10.7759/cureus.80691

**Published:** 2025-03-16

**Authors:** Sakiko Honda, Michiyo Yamano, Tatsuya Kawasaki

**Affiliations:** 1 Department of Cardiology, Matsushita Memorial Hospital, Moriguchi, JPN

**Keywords:** auscultation, diagnosis, patent ductus arteriosus, physical examination, systolic murmur

## Abstract

Patent ductus arteriosus (PDA) is a congenital anomaly characterized by a persistent connection between the descending aorta and the pulmonary artery. While patients with PDA typically present with a continuous murmur, atypical cases, such as those with systolic murmurs or no murmurs (although the exact mechanism remains unclear), pose diagnostic challenges. We report a case of a 38-year-old woman with alcoholic liver cirrhosis, referred for liver transplantation, in whom a systolic ejection murmur prompted further evaluation. Diagnostic imaging and right heart catheterization confirmed PDA with mild pulmonary hypertension, without evidence of Eisenmenger physiology. This case underscores the importance of recognizing atypical murmur patterns in the diagnosis of PDA to avoid misdiagnosis or delayed diagnosis.

## Introduction

Patent ductus arteriosus (PDA) is defined as a persistent connection between the descending aorta and the pulmonary trunk, with an estimated incidence of approximately one in 2,000 births [1]. This essential fetal structure typically closes after birth, usually within 48 hours, but persistence of ductal patency beyond the first few weeks of life is considered abnormal and leads to pulmonary overcirculation and left heart volume overload due to left-to-right shunting [1]. Although the incidence may vary depending on the age at the time of the study (e.g., much higher in preterm infants), the presence of a continuous murmur is extremely helpful in identifying this condition. However, patients with PDA without a continuous murmur (referred to as atypical or silent PDA) may also present, partly due to the effects of pulmonary hypertension and coexisting conditions [2]. Here, we report a case of alcoholic liver cirrhosis in which a systolic ejection murmur triggered the diagnosis of PDA.

## Case presentation

A 38-year-old woman was referred to the cardiology department of our hospital for cardiac screening prior to allogeneic liver transplantation. Her medical history was notable for alcoholic liver cirrhosis, hepatic encephalopathy, adjustment disorder, anorexia nervosa, and hypertension. Her medications included amlodipine, ursodeoxycholic acid, spironolactone, camostat mesylate, rifaximin, a branched chain amino acids rich soft powder nutrient mixture (Aminoleban EN®, Otsuka Pharmaceutical Co., Tokyo), lactulose, and magnesium oxide. She quit smoking eight years earlier after a 0.5 pack-year history, stopped drinking several months earlier after consuming more than 100 grams of ethanol daily for years, and had no known allergies. There was no family history of cardiovascular disease.

**Figure 1:**
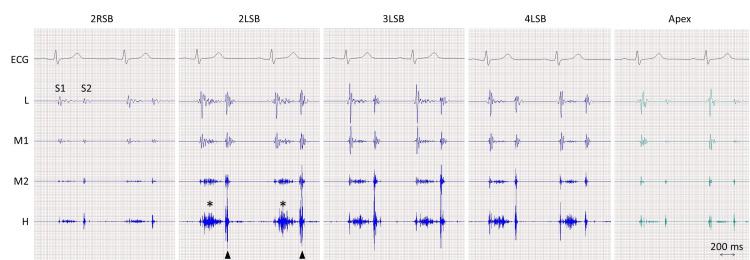
Phonocardiography. A systolic ejection murmur (asterisks) is best seen at the second left sternal border (LSB). The pulmonary component of the second sound (S2) is louder than the aortic component (arrowheads). ECG, electrocardiography; H, high frequency; L, low frequency; M1, lower-middle frequency; M2, higher-middle frequency; RSB, right sternal border; S1, the first sound.

On examination, she was oriented. Her blood pressure was 97/71 mmHg, her pulse rate was 60 beats per minute, and her oxygen saturation level was 96% while breathing ambient air. The jugular venous pulsation was not elevated. Cardiac auscultation revealed a split-second sound with an increased pulmonary component and a systolic ejection murmur in the pulmonary region (Figure 1), findings typical of atrial septal defect with pulmonary hypertension (PH). Both lungs were clear on auscultation and there was no edema in the legs.

Electrocardiography was normal and a chest radiograph showed mild cardiomegaly with dilated pulmonary arteries in the lack of pulmonary congestion or pleural effusion. Her complete blood cell count showed a hemoglobin of 6.3 g/dl and a platelet count of 51,000 per microliter. The levels of bilirubin, aspartate aminotransferase, and alanine aminotransferase, were 10.5 mg/dl, 80 U/l, and 81 U/l. The prothrombin time-international normalized ratio, activated partial thromboplastin time, and ammonia level were 1.53, 69.7 s, and 116 μg/dl (reference value, ≤65). The brain natriuretic peptide level was elevated to 218.4 pg/ml (reference value, ≤18.4). Other laboratory data are shown in Table 1.

**Table 1:** Laboratory data. eGFR, estimated glomerular filtration rate.

Variable	Reference range	On presentation
White blood cell count (/μl)	3,300-8,600	3,300
Hemoglobin (g/dl)	11.6-14.8	6.3
Mean corpuscular volume (fl)	83.6-98.2	96.3
Platelet count (/μl)	158,000-348,000	51,000
Partial thromboplastin time (s)	11-14	17.1
International normalized ratio	0.8-1.2	1.53
Activated partial thromboplastin time (s)	28-38	69.7
Total bilirubin (mg/dl)	0.4-1.5	10.5
Direct bilirubin (mg/dl)	≤0.3	6.3
Aspartate aminotransferase (U/l)	13-30	80
Alanine aminotransferase (U/l)	7-23	81
Lactate dehydrogenase (U/l)	124-222	391
Alkaline phosphatase (U/l)	38-113	142
Total protein (g/dl)	6.6-8.1	5.0
Albumin (g/dl)	4.1-5.1	2.0
Sodium (mmol/l)	138-145	137
Potassium (mmol/l)	3.6-4.8	5.4
Urea nitrogen (mg/dl)	8-20	30
Creatinine (mg/dl)	0.46-0.79	0.89
Creatine kinase (U/l)	41-153	15
C-reactive protein (mg/dl)	0.00-0.14	0.13
Blood sugar (mg/dl)	73-109	133
eGFR (ml/min/1.73 m^2^)	≥60	57.3
Ammonia (μg/dl)	≤65	116
Brain natriuretic peptide (pg/ml)	≤18.4	218.4

**Figure 2:**
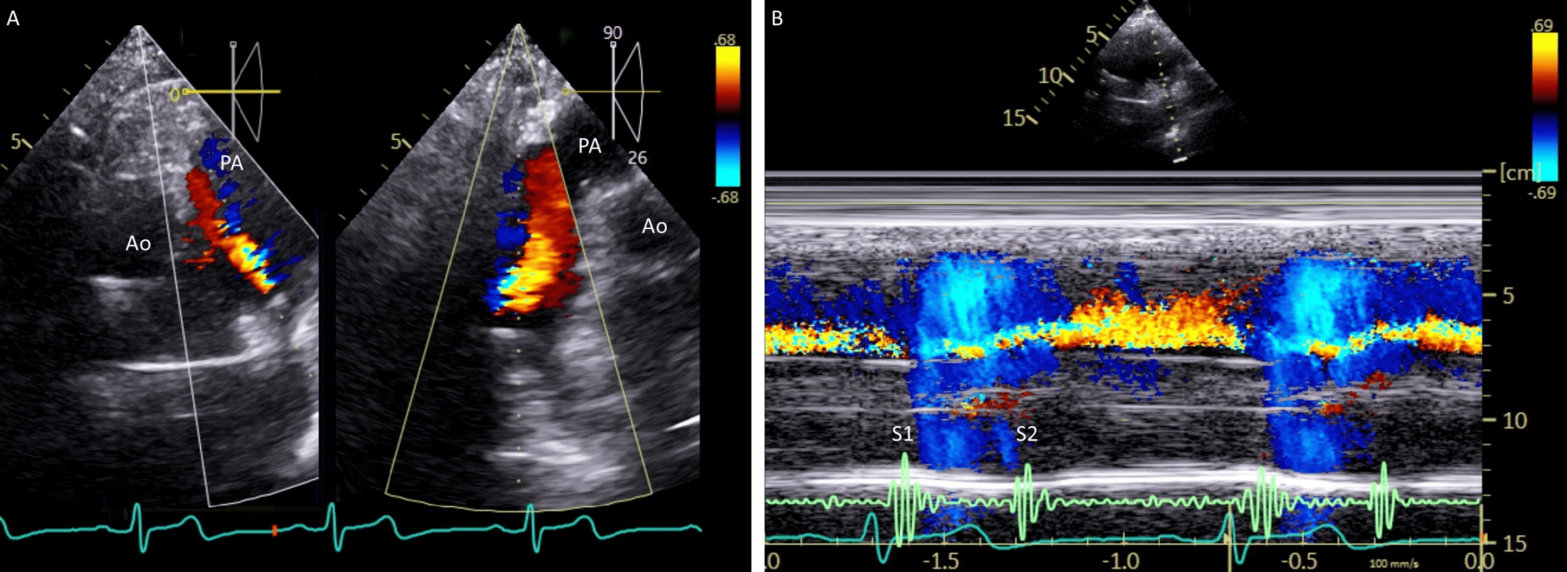
Echocardiography. Short-axis images (Panel A, left) at the level of the aorta (Ao) and its 90-degree rotated view (Panel A, right) show flow directed toward the pulmonary artery (PA). Note that, on the color M-mode (Panel B), the flow, which begins at the first heart sound (S1), persists through and beyond the second heart sound (S2).

Transthoracic echocardiography showed a left ventricular ejection fraction of 66% with normal ventricular dimensions. Doppler imaging revealed mild tricuspid regurgitation and the systolic pulmonary artery pressure was estimated to be 41 mmHg without right heart enlargement. A continuous flow toward the pulmonary valve was unexpectedly noted in the main pulmonary artery (Figure 2), findings consistent with PDA. Contrast echocardiography with an agitated saline was negative.

**Figure 3:**
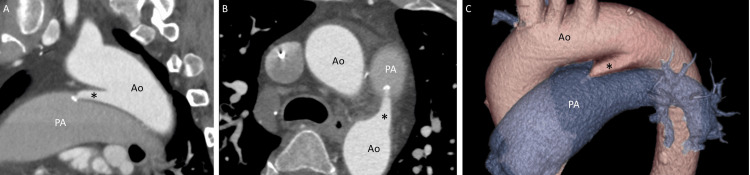
Computed tomography. A direct connection (asterisks) between the aorta (Ao) and the pulmonary artery (PA) is observed, with a small calcification at its exit to the PA, as seen in the sagittal image (Panel A), short-axis image (Panel B), and three-dimensional reconstruction (Panel C).

Computed tomography of the heart after the administration of contrast material showed a direct connection (6.6 mm in diameter and 23.0 mm in length) from the descending aorta to the main pulmonary artery (Figure 3). A diagnosis of PDA was made.

Cardiac catheterization showed normal coronary arteries, a mean pulmonary capillary wedge pressure of 6 mmHg, pulmonary artery pressure of 28 mmHg, cardia index of 4.7 l/min/m^2^ (reference value, 2.3 to 4.2), pulmonary vascular resistance of 1.4 to 2.1 Wood Unit (reference value, <3.0), and a ratio of pulmonary to systemic blood flow (Qp/Qs) of 1.4 with an oxygen saturation step-up from 61% in the right ventricle to 70% in the main pulmonary artery.

A diagnosis of PDA with PH was made. The patient underwent liver transplantation at another hospital and the clinical course was reportedly uneventful.

## Discussion

The current patient was found to have a heart murmur during the examination for liver transplantation due to alcohol liver cirrhosis. She showed systolic ejection murmur best heard at the pulmonary area, in the absence of diastolic murmur, both on auscultation and on phonocardiography, although continuous shunt flow was confirmed not only systole but also diastole on echocardiography. Furthermore, given the increased pulmonary component of the second sound, the patient was thought to have PH, which was confirmed by invasive cardiac catheterization.

The presence of continuous murmur, often termed a machinery or grinding murmur, due to pressure gradient between the aorta and the pulmonary artery throughout the whole cardiac cycle is the most important clinical feature for symptomatic PDA [3]. Continuous murmur can also be the first clue to the diagnosis of PDA because this condition is usually isolated and a small to moderate degree of shunt is unlikely to become symptomatic [4]. Thus, physical examination has played a pivotal role in recognizing the presence of PDA in clinical practice although it is not always true that all abnormal flow produces murmurs without intraobserver variations. It is important to emphasize that a continuous murmur is defined as one that remains unchanged in characteristics before and after the second heart sound and should not be mistaken for the combination of systolic and diastolic murmurs, commonly referred to as a to-and-fro murmur. A to-and-fro murmur changes its auscultatory features over the second heart sound, such as a systolic ejection murmur due to aortic stenosis and a diastolic murmur due to aortic regurgitation.

Causes of systolic ejection murmurs include aortic stenosis, aortic sclerosis, pulmonary stenosis, and left ventricular outflow tract obstruction, none of which were observed in this patient. Non-cardiac conditions such as severe anemia as observed in the present patient, fever, thyrotoxicosis, pregnancy, and vitamin B_1_ deficiency may also cause systolic ejection murmurs. Based on the phonocardiographic characteristics of the murmur (i.e., best heard in the pulmonary area) and the shunt flow from the aorta to the pulmonary artery observed on echocardiography, it is reasonable to conclude that the systolic ejection murmur was associated with PDA.

The exact mechanism of the systolic ejection murmur in this patient remains unclear. However, it is plausible that the hyperdynamic status resulting from anemia and liver cirrhosis contributed to pulmonary hypertension, altering the typical continuous murmur of PDA into an atypical presentation. In cases where aortic and pulmonary artery pressures equilibrate, as seen in patients with Eisenmenger PDA, murmurs may disappear entirely, resulting in a silent PDA [5]. In this patient, Eisenmenger PDA was ruled out due to the presence of only mild pulmonary hypertension and the absence of increased pulmonary vascular resistance. Interestingly, six patients with PDA and equal pulmonary arterial and aortic pressures have been reported to exhibit only a diastolic murmur [6].

Continuous murmurs are caused by continuous blood flow shunting from high-pressure or high-resistance circulation to low-pressure or low-resistance circulation, persisting throughout systole and diastole without interruption, including the second heart sound [7]. The underlying conditions include aortic-pulmonary communications (e.g., PDA), arteriovenous communications (e.g., coronary arteriovenous fistulas and ruptured sinus of Valsalva aneurysms into right-sided heart chambers), and alterations in arterial and venous flows (e.g., venous hum, mammary soufflé, and hyperthyroidism). It is noteworthy that PDA murmurs in children may present as systolic murmurs due to pulmonary vasoconstriction secondary to a large shunt. This often results in a moderate degree of pulmonary hypertension, which reduces the aortic-to-pulmonary artery pressure gradient more during diastole than systole. Although the present patient exhibited a systolic ejection murmur, PDA murmurs can also be pansystolic, which may lead to misdiagnosis or delayed diagnosis [8].

The present patient exhibited pulmonary hypertension, likely due to alcoholic liver cirrhosis. On echocardiography, flow was observed even during diastole; however, it is reasonable to consider that the pressure gradient was insufficient to produce an audible murmur. PDA murmurs can be atypical or silent, depending on the direction of the jet within the pulmonary artery. Bennhagen et al. reported that, in 14 out of 15 children with silent PDA, the ductal flow did not contact and remained away from the anterior wall of the main pulmonary artery [9]. In our patient, the shunt flow was directed toward the anterior wall of the main pulmonary artery, although no direct contact was suspected. Importantly, their study also found no correlation between the presence of a murmur and the size of the arterial duct.

## Conclusions

This case report emphasizes the importance of recognizing not only continuous murmurs but also various murmur patterns in patients with PDA, as misdiagnosis or delayed diagnosis may occur without careful physical examination, even in the era of advanced imaging techniques, although physical examination should guide suspicion and prompt appropriate imaging, rather than serving as the sole diagnostic tool.
